# Measuring decision aid effectiveness for end-of-life care: A systematic review

**DOI:** 10.1016/j.pecinn.2024.100273

**Published:** 2024-03-13

**Authors:** M. Courtney Hughes, Erin Vernon, Chinenye Egwuonwu, Oluwatoyosi Afolabi

**Affiliations:** a School of Health Studies, Northern Illinois University, Wirtz Hall 209, DeKalb, IL 60115, USA; b Department of Economics, Seattle University, Pigott 522, Seattle, WA 98122, USA

**Keywords:** End-of-life care, Decision aids, Systematic review, Advance care planning, Palliative care, Dying

## Abstract

**Objective:**

To systematically review research analyzing the effectiveness of decision aids for end-of-life care, including how researchers specifically measure decision aid success.

**Methods:**

We conducted a systematic review synthesizing quantitative, qualitative, and mixed-methods study results using Preferred Reporting Items for Systematic Reviews and Meta-Analysis guidelines. Four databases were searched through February 18, 2023. Inclusion criteria required articles to evaluate end-of-life care decision aids. The review is registered under PROSPERO (#CRD42023408449).

**Results:**

A total of 715 articles were initially identified, with 43 meeting the inclusion criteria. Outcome measures identified included decisional conflict, less aggressive care desired, knowledge improvements, communication improvements, tool satisfaction, patient anxiety and well-being, and less aggressive care action completed. The majority of studies reported positive outcomes especially when the decision aid development included International Patient Decision Aid Standards.

**Conclusion:**

Research examining end of life care decision aid use consistently reports positive outcomes.

**Innovation:**

This review presents data that can guide the next generation of decision aids for end-of-life care, namely using the International Patient Decision Aid Standards in developing tools and showing which tools are effective for helping to prevent the unnecessary suffering that can result when patients' dying preferences are unknown.

## Highlights

• End-of-life care decision aid use consistently reports positive outcomes.
• International Patient Decision Aid Standards enhance end-of-life care decision aid.
• End-of-life care decision aids provide value across different countries.

## 1 Introduction

End-of-life care refers to the medical care and support provided during the days, weeks, or even months surrounding death. Often, individuals nearing the end of their lives experience unaddressed symptoms and requirements, including pain, debility, emotional anguish, and anxiety [1]. Some goals of quality end-of-life care that patients have identified include adequate pain control, preventing undue prolongation of dying, relieving concerns, and improving relationships with loved ones [1,2]. The end-of-life stage also involves making substantial decisions that impact patients, their caregivers, and healthcare workers. This stage has been described as stressful for patients due to inadequate preparation, with healthcare workers sometimes making decisions for their patients [3]. The fear of causing distress for patients, feeling unprepared, and being unsure of prognosis have been identified as barriers healthcare workers face in discussing end-of-life care with patients [4]. From the patient's perspective, this has resulted in inconsistency with the goals of care, reflecting the crucial value of effective decision making in end-of-life care [5].

Advance care planning (ACP) involves discussions between patients and their care providers about their preference for care in the near-term and future, including decisions to make in case of possible deterioration that might limit their decision making capacity [6]. Using decision aids to assist with medical decision making has been found to help improve the quality of end-of-life care among older patients [7]. Decision aids are customized tools developed to guide patients in making informed decisions regarding treatment or diagnostic options that allow for considering possible benefits or harms. Compared to routine decision making, they have the added benefits of improving the patient's knowledge of treatment or diagnostic options, reducing the burden of decision making, increasing the patient's participation in making decisions, creating risk perceptions of benefits and harms, and reducing decisional conflicts [3,[7], [8], [9]]. Yet, despite these benefits, a systematic review showed that decision aids may be insufficient in addressing patient's needs [6].

Previous systematic reviews on decision aids have evaluated their effectiveness in supporting shared decision making for treatment options for specific diseases [[10], [11], [12]] or treatment in general [7] or limited their scope to contemporaneous decision-making in end-of-life care and did not include advance care planning [6]. There is a gap in examining the effectiveness of decision aids for end-of-life care that is not limited to a specific disease or a dying population. Thus, there is limited holistic knowledge about the effectiveness of decision aids for end-of-life care. Such knowledge would inform researchers and practitioners who are studying and developing tools for general end-of-life care decision making. We aim to provide the first systematic review of the literature to date focusing on the effectiveness of decision aids for end-of-life care across populations with and without disease who may or may not be dying. Such decision aids may include guidance on palliative care and/or hospice options, help with advance directive decision making, or provide guidance on both.

## 2 Methods

### 2.1 Search strategy

The systematic review is registered at PROSPERO (CRD 42023408449). We performed full-length database searches on articles published through February 18, 2023 using the Preferred Reporting Items for Systematic Reviews and Meta-Analyses (PRISMA) guidelines (14). We explored the databases of Pubmed, CINAHL, Proquest Federated and PsycInfo. The keyword strings used to capture relevant studies were “decision aid” or “decision tool” along with any of the following: “terminal care,” “caregiver*,” “bereave*,” “inpatient,” “attitude to death,” “end of life,” “hospice*,” “terminally ill,” “palliative*,” “advance care,” “advanced,” “morphine and cancer,” or “cancer pain.” (Note that “*” represents any group of characters, including no character.) We initially included peer-reviewed scholarly literature that contained these keywords in either the title or the abstract.

### 2.2 Inclusion /exclusion criteria

To be included, the articles must have examined the use of decision aids at the end of life.The researchers aimed to capture research that focused on decision aids tailored for individuals either entering the end-of-life care stage or those explicitly interested in planning for such a time. As such, the decision aids included in the articles could have different focuses, including making emergent hospice and palliative care decisions or preparing for advance directives related to future care. We included articles related to disease-specific decision aids if they examined a decision aid specifically related to an end-of-life care focus. Both qualitative and quantitative analysis articles were included.

Duplicates were excluded. Articles that were not focused on end-of-life, not evaluative, not focused on decision aids, and not in English were excluded. Review articles were also excluded.

### 2.3 Extraction process

At least two authors independently examined the titles and abstracts of every study that met the inclusion criteria. Disagreements were resolved by accessing the full-text articles. At least two authors independently read the full-text articles to determine study inclusion. Any disagreements were resolved by discussion among the four authors.

At least two authors independently extracted key information from all articles, including the decision aid name, decision aid description, decision aid format, study population disease focus, country, study design, outcomes measured, results, and whether the International Patient Decision Aid Standards (IPDAS) [13] were used in the decision aid development. One way to help ensure better quality (e.g., realistic expectations, values-choice agreement, knowledge [14]) for patient decision aids is for individuals and groups developing and evaluating decision aids to use the framework and 64-item checklist provided by the International Patient Decision Aid Standards (IPDAS) Collaboration [13]. This collaboration was established 20 years ago to guide researchers, practitioners, and other stakeholders in designing and improving patient decision aids. We assessed article quality using the Mixed Methods Appraisal Tool version 2018 [15]. This tool appraises the quality of the methods used for qualitative, quantitative, and mixed method studies. Studies were rated as low, medium or high based on the criteria outlined in the tool. Studies were of low-quality if 75% of the methodology criteria were met, of medium-quality if 76%–86% of the methodology criteria were met and of high-quality if above 86% of the criteria were met [16]. Disagreements were resolved via deliberations among the four researchers.

## 3 Results

### 3.1 Search results

**Fig. 1:**
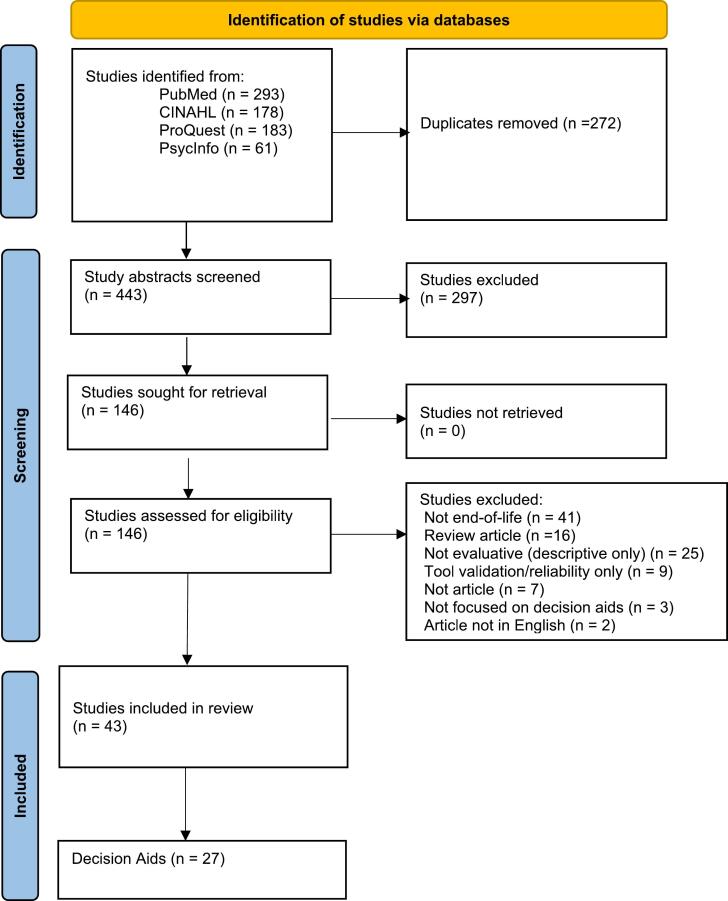
PRISMA flow diagram.

We initially retrieved a total of 715 articles from PubMed, CINAHL, ProQuest Federated, and PsycInfo. After removing duplicates, we screened 443 abstracts and reviewed 146 articles in full. Fig. 1 presents our selection process following PRISMA guidelines. Table 1 presents the study characteristics for the 43 studies we included after exclusions. More detailed information about each study is in Supplementary Tables 1 and 2.

**Table 1:** Characteristics of the studies (*n* = 43).

	Number of studies
Quantitative study quality (*n* = 39)⁎	
High	20 (51.3%)
Medium	12 (30.8%)
Low	7 (17.9%)
Qualitative study quality (*n* = 9)⁎	
High	8 (88.9%)
Medium	0 (0%)
Low	1 (11.1%)
Countries⁎⁎	
USA	32 (74.4%)
South Korea	2 (4.7%)
Canada	2 (4.7%)
Taiwan	2 (4.7%)
Japan	2 (4.7%)
Germany	1 (2.3%)
Korea	1 (2.3%)
Netherlands	1 (2.3%)
France	1 (2.3%)
Decision aid format	
Video	15 (34.9%)
Online (static)	10 (23.2%)
Online (interactive)	7 (16.3%)
Video and booklet	5 (11.6%)
Paper	3 (7.0%)
Interview	2 (4.7%)
Video and interview	1 (2.3%)
Disease focus	
General population	15 (35.0%)
Cancer	10 (23.3%)
General, terminally ill	9 (20.9%)
Dementia	5 (11.6%)
Advanced liver disease	1 (2.3%)
Chronic obstructive pulmonary disease	1 (2.3%)
End stage renal disease	1 (2.3%)
High-risk surgery	1 (2.3%)
Focus areas (study type)⁎⁎⁎	
Decisional conflict (quant = 13, qual = 1, mixed = 3)	17 (39.5%)
Knowledge improvements (quant = 10, qual = 2, mixed = 3)	15 (34.9%)
Communication improvements (quant = 9, qual = 1, mixed = 5)	15 (34.9%)
Less aggressive care desired (quant = 10, qual = 1, mixed = 3)	14 (33.0%)
Tool satisfaction (quant = 5, qual = 2, mixed = 1)	8 (18.6%)
Less aggressive care action completed (quant = 6, qual = 0, mixed =0)	6 (14.0%)
Patient anxiety or well-being (quant = 4, qual = 0, mixed = 1)	5 (11.6%)

*Notes*: quant = quantitative; qual = qualitative.

⁎ Mixed methods studies included in both quantitative and qualitative sections.

⁎⁎ One study took place in both the USA and Japan so the percentages do not add up to 100%.

⁎⁎⁎ Section totals do not equate to total studies due to multiple examined outcomes within studies.

The studies were conducted in 14 countries, with about three-fourths from the United States. All countries included in the studies have high-income economies based on World Bank classifications [17]. The majority of the studies (over 80%) were of either medium or high quality. Decision aids were publicly accessible for 37% of the decision aids mentioned in the articles and are shown in Table 2, which presents all decision aids found in the studies. (Note: we counted each decision aid only once, even if it appeared in more than one reviewed article.) Of the 27 decision aids studied, one-third (9) mentioned using IPDAS standards within the tool development and two-thirds (6) of those using IPDAS reported positive outcomes. Nearly half of the decision aids were video-based and two-thirds were web-based. The decision aids in the study were either geared toward the general population (35%), those with a terminal illness diagnosis (21%), or a specific terminal diagnosis such as advanced cancer or dementia (44%). We present the following results by the outcome focus area of the decision aids in the studies.

**Table 2:** Description of decision aids.

Decision Aid	Description	Provider Type and Role	Website	IPDAS⁎	Studies and Outcome Success⁎⁎
Advance Care Planning for Seniors	A booklet with cartoons intended to educate older adults and their surrogates about advance care planning.	N/A	N/A	N/S	Ke (2021) [Y]
Advance care planning video (no official name)	5-min video that introduces choices for medical care at the end of life that included visual images	N/A	N/A	Y	Ufere (2022) [Y]
Advance directive for artificial nutrition and hydration	Video-based tool which presented possible harms and benefits of artificial nutrition and hydration as well as a value clarification exercise.	N/A	N/A	Y	Friend (2021) [Y]
Choice Help (“Kkeuzehulppz”)	Interactive short videos portraying actors discussing their chosen locations of death and their associated values. Depending on users' selected values, certain videos were played (two with location supporting their values and one with location that does not match values).	N/A	https://www.dz.nl/patient/keuzes-rond-het-levenseindeN/A	N/S	Kerstholt (2012) [Y]
CPR-VDA	7-min video designed for participants to view independently on a portable screen. It includes information about CPR, the alternative option of comfort care, and information about the patient experience and health outcomes.	N/A	A Decision Aid to Prepare Patients And Their Families For Shared Decision-Making About Cardio-Pulmonary Resuscitation (CPR) on Vimeo	N/S	Kapell Brown (2018) [Y]
Decision Aid Form (DAF) (no official name)	1-page decision aid for physicians to stratify hospitalized patients for care	Sheet filled out by provider during admission, consultation, transfer or change of clinical state.	https://cdn.amegroups.cn/static/magazine_modules/imgRender/dist/index.html?imgSource=https://cdn.amegroups.cn/journals/amepc/files/journals/8/articles/90359/public/90359-PB6-9674-R1.jpg	N/S	Vigouret-Viant (2022) [M]
DecisionKEYS for Balancing Choices: Cancer Care	Multicomponent program intended to improve decision making skills when there are multiple complex and stressful choices, help with a specific decision, and provide structured time for support by healthcare providers for decision making	Patients and decision partners complete the decision aid in a clinical setting just prior to provider conversation.	N/A	N/S	Jones (2018) [Y]
Four Conversations	An online and personalized coping and decision aid curriculum, on the completion of advance care directives and shared decision making among patients and their loved ones, clinicians, and spirit. “Four [online] modules, each of which consists of a series of interactive videos and workbook activities focusing on EOL reflections and wishes for ‘how one wants to live and die.’ The web-based videos comprised a therapist conversing with a patient around EOL and exercises such as worksheets, visualizations, and tools were available to the participants to access at any time”	Participants complete the activities in each module, and then communicate with a specially trained “Pillar Guide” by e-mail and/or telephone to discuss what they have learned over a four-week period.	N/A	N/S	Smith (2020) [N]
Goals of Care	18-min video, designed for surrogate decision makers of nursing home patients, that uses patient stories, balanced presentation, and simple language to enable comprehension at an 8th grade educational level. Content includes information about advanced dementia, role of the surrogate decision-maker, treatment goals, and treatment options consistent with each goal. Also included print handout (Einterz)	After video, a structured meeting between the surrogate and the interdisciplinary care plan team at the nursing home occurs within the next 3 months	N/A	Y	Einterz (2014) [Y], Hanson (2015) [Y]
InformedTogether	Web-based decision aid designed to support shared advance care planning between severe COPD patients and their doctors.	Intended to be used by provider and patient together during clinic visit	N/A	Y	Uhler (2015) [M]
Living with Metastatic Breast Cancer: Making the Journey Your Own’	30-min video and booklet decision aid aimed “to help women learn about the disease and the options for treatment and to communicate their preferences and goals for treatment to providers and family members. The decision aid follows four women living with metastatic breast cancer and depicts how they live with the diagnosis, how they work with their doctors to make decisions, and how they continue to have hope”	N/A	N/A	N/S	Ozanne (2009) [N]
Looking Ahead: Choices for Medical Care When You're Seriously Ill	37-min DVD and 51-page booklet to encourage conversations, advance care planning and patient-centered decision making related to advanced illness	N/A	N/A	N/S	Bakitas (2017) [Y], Jones (20,150 [M]), Matlock (2014) [M}]
Making Your Wishes Known: Planning Your Medical Future	1–2 h comprehensive computer-based educational program designed to facilitate ACP discussions between patients and their medical team	N/A	https://vitaldecisions.net/solutions/my-living-voice/	N/S	Green (2009) [Y], Green (2015) [M[, Green (2020) [Y], Hossler (2011), Markham (2015) [Y[, Levi (2017) [M], Lipnick (2020) [M], Schubart (2019) [N], Simmons (2022) [M], Thiede (2021) [M], Van Scoy (2016) [Y]
Patient decision aids before high risk surgery (no official name)	2 booklets a patient could write in to 1) consider a treatment preference and communicate it to a surrogate decision-maker or health care provider, and 2) decide to continue or stop treatment with the hope of prolonging life if recovery becomes difficult	N/A	https://bmcpalliatcare.biomedcentral.com/articles/10.1186/s12904-022-01068-2/tables/1	Y	Yamamoto (2022) [Y]
Patients Want to Know the Truth/Advance Care Planning	20-min decision support video on a notebook computer and a 43-page book on advance care planning. Advance Care Planning is the use of the same material modified for the general population (versus advanced cancer population).	N/A	N/A	Y	Kang (2020) [Y],Yun (2011) [M], Yun (2019), [Y]
Person-Centered Oncologic Care and Choices (P-COCC)	Video decision aid on end-of-life care options and an interview eliciting patient values regarding their goals, concerns, and support systems for oncology patients	Interviewer trained in cognitive interviewing and serious illness research conducted value-focused patient interview	N/A	N/S	Agarwal (2020) [Y]
Plan Well Guide	Online interactive program with paper-based values clarification form that generates a personalized “Dear Doctor” letter recording the nature of the conversation, the stated values, and expressed treatment preferences	Trained facilitator walks patient through guide and works with patient to complete “Dear Doctor” letter	https://planwellguide.com/healthcare-professionals/#:~:text=Plan%20Well%20Guide%20can%20help,of%20your%20time%20and%20energy.	Y	Heyland (2020) [M]
PREPARE plus Advance Directive	Web-based guide which uses video stories, modeling of behaviors, and a five-step process. Goals are “to motivate and prepare individuals to discuss their values and care preferences and, using behavior change techniques, help individuals move along the ACP behavior change pathway.” An easy to read advance directive form was included.	N/A	www.prepareforyourcare.org	N/S	Lum (2018) [Y]
PRT Video Tool (no official name)	10-min video tool with four segments explaining: the process of radiation simulation, what to expect at the time of treatment;, common side effects, and the purpose of palliative care.	N/A	https://www.mskcc.org/videos/palliative-radiation-therapy	Y	Dharmarajan (2019) [M]
Supportive and Palliative Care Indicators Tool (SPICT-DE™)	Online information sheet supporting primary care physicians in the identification of patients with deteriorating health and potentially unmet palliative care needs	Physician used tool	https://www.spict.org.uk/	N/S	van Baal (2022) [Y]
Tables of information (no official name)	Tables of information which provides a concise review of diagnosis, prognosis, treatment options, side effects, and when to call the doctor	N/A	N/A	N/S	Smith (2011) [Y]
Video support tool of advanced dementia (no official name)	2-min video support tool of advanced dementia patient and verbal narratives about advanced dementia and goals of care	Person (position not stated) to read narrative	https://www.bmj.com/content/338/bmj.b2159	N/S	Volandes (2009) [Y], Volandes (2010) [Y]
Video support tool of advanced dementia (no official name)	6-min video support tool of advanced dementia patient and verbal narratives about advanced dementia and goals of care	Person (position not stated) to read narrative	N/A	N/S	Volandes (2011) [Y]
Video portraying choices of health care in advanced cancer (no official name)	6-min video portraying three choices of health care in advanced cancer: life-prolonging care, basic medical care and comfort care	N/A	N/A	N/S	Volandes (2012) [M]
Video regarding physician orders for life-sustaining treatment (no official name)	7-min video decision aid for the Cardiopulmonary Resuscitation and Medical Interventions sections of the West Virginia version of Physician Orders for Life-Sustaining Treatment	N/A	N/A	Y	Gallegos (2020) [Y]
Video regarding CPR and intubation (no official name)	3-min digital video regarding CPR and intubation played on an iPad at patient's bedside	Physician not on patient's care team verbally communicated participants' post-video CPR and intubation preferences to at least one physician on patient's care team after decision aid was used	https://www.acpdecisions.org/⁎⁎⁎	N/S	El-Jawahri (2015) [Y]
Virtual reality video and handout (no official name)	6-min virtual reality video presenting a first-person perspective of a patient with chronic obstructive pulmonary disease (COPD) to allow participants to immerse themselves in the complete clinical process of typical end-of-life care, starting with CPR in the intensive care unit, followed by withdrawn LST, hospice ward care, and hospice home care.	N/A	N/A	N/S	Hsieh (2020) [Y[

⁎ The International Patient Decision Aid Standards (IPDAS) was used in development.

⁎⁎ For Y = yes; N = no; M = mixed qual = qualitative.

⁎⁎⁎ A code from a patient's healthcare provider is required to access the decision aid. Therefore, this decision aid is not considered publicly accessible.

### 3.2 Decisional conflict

Decisional conflicts refer to internal struggles or uncertainty that patients or their families face when making choices related to end-of-life treatment options [18] and were examined in about 40% of the studies. Ten out of 13 of the quantitative studies examining such conflicts found positive outcomes from decision aid use. The results from the studies reporting positive decisional conflict outcomes were all from the patient's perspective and varied in characteristics such as country of origin, tool format, length, disease focus, and whether IPDAS were used during development. One quantitative study reported a reduction in patient reported decisional conflict after decision aid use but no reduction from the provider perspective [19]. The 4 qualitative studies that reported decisional conflict themes were all from the United States and consistently reported positive results [[20], [21], [22], [23]].

### 3.3 Knowledge improvements

Knowledge improvements about end-of-life care refer to enhancing one's understanding about the medical, emotional, and practical aspects of nearing the end of life. This knowledge can help individuals make more informed decisions [24]. Nine of the 10 quantitative studies examining knowledge improvements reported positive results. Four United States quantitative studies noted improved ACP knowledge for patients using the Making Your Wishes Known decision aid [[25], [26], [27], [28]]. Three other United States quantitative studies also reported increased knowledge after the use of video decision aids, including reported improved goals of care knowledge after patients viewed a 6-min decision aid video geared toward advanced cancer patients [29], a better understanding of Physician Orders for Life-Sustaining Treatment form options after a 7-min decision aid video on the topic [30], and improved treatment knowledge after a video specific to palliative radiation therapy [31]. Only one quantitative study reported no knowledge improvements associated with decision aid use: a US study of a 37-min decision aid DVD and associated booklet reported no improvements to patient advance directive knowledge [32].

### 3.4 Communication improvements

Communication improvements refer to enhancing the quality and effectiveness of exchanging information, ideas, and feelings between patients, their family, and their healthcare providers [33]. Of the 9 quantitative studies that examined communication improvements, 5 focused on the interaction between patients and healthcare providers. Two of these studies, one from Canada [19] and one from the United States [28], reported decision aids improved concordance between patients and providers, while two other of these studies from the United States reported no improvements in patient and provider communication after patient decision aid usage [34,35]. One United States quantitative study examined a decision aid specifically designed for family or surrogate caregivers of nursing home patients and found patient and provider improvements in long-term goal concordance [36]. Note that a surrogate caregiver is someone appointed to make medical decisions on behalf of a patient who is unable to do so. Three qualitative studies touched on themes of provider and patient relationships, with two noting potential improvements after using different tools in the United States [22] and Japan [37]. The decision aid *Making Your Wishes Known* had positive and neutral outcomes regarding patient and provider communication, depending on the study, two of which were quantitative [28,35] and one of which was qualitative [20].

The studies examining communication between patients and their family members or surrogates mainly reported positive outcomes. However, of the 4 quantitative studies in this category, one was a randomized control trial of a Korean decision aid that found that it did not change the decision to discuss the terminal diagnosis with loved ones [38]. Additionally, a quantitative study of a United States decision aid reported mixed results in this area noting that while there were improvements in conversations with the patient and medical team, there was no change in surrogates' preparedness for decision making [23]. Qualitative studies examining patient and surrogate communication themes reported positive findings [21,23,37].

### 3.5 Less aggressive care desires and actions

Less aggressive care desires refer to preferences for receiving comfort and management of pain and symptoms rather than life-sustaining medical interventions and treatment that often have burdensome side effects and cause high discomfort [39]. Of the 10 quantitative studies examining the topic, all found that decision aids led to patients wanting less aggressive care. Studies of five different video decision aids in the United States associated the aids with less desire for CPR [29,40,41], artificial hydration therapy [42], or aggressive care in general [29,43]. Decision aid studies outside of the US reported similar findings, with South Korea [44], Canada [45], Taiwan [46], and Japan [42] studies all reporting less aggressive care desired after decision aid use. Four qualitative studies, all examining various US decision aids, echoed most quantitative research with themes about patients desiring less aggressive care after decision aid exposure [22,23,47,48].

The 6 quantitative studies examining decision making action outcomes related to end-of-life care experienced more mixed results. For example, while the patients in two different studies expressed more of a desire for less aggressive care, there was no increase in documented do-not-resuscitate orders [29] or observed pursuance of less aggressive care [49]. Another United States quantitative study that reported increased knowledge of treatment options did not find changes to palliative care consultations [31]. Three other United States quantitative studies did report positive actions toward less aggressive care with increased orders to withhold CPR and intubation [40], documented ACP [50] and the number of life-sustaining medical treatments after decision aid use [51].

### 3.6 Patient anxiety or well-being

Patient anxiety refers to a state of uneasiness or fear related to their health condition or the healthcare system. In contrast, patient well-being refers to a positive state of mind, functioning, and satisfaction [52]. Four quantitative studies and 1 mixed study examined decision aid impact on patients' anxiety levels or well-being. In their quantitative examination of the video and book-based decision aid *Patients Want to Know the Truth/Advance Care Planning*, Yun and colleagues [38] found the tool positively impacted patients' mood and general wellbeing. A quantitative study in Germany also observed patient quality of life improvements with decision aid use [53]. In contrast, 2 United States quantitative studies did not find decision aid use improved patient anxiety [25,28], and 1 United States mixed methods study did not find decision aid use improved general stress levels [21].

### 3.7 Tool satisfaction

While not a direct measure of patient outcomes, tool satisfaction was a common measurement across studies. Tool satisfaction refers to the level of fulfillment of one's expectations or needs that individuals experience from using the decision aids. The 8 studies measuring tool satisfaction (5 quantitative, 2 qualitative, and 1 mixed) were all from the United States, reporting positive patient satisfaction [27,41,47,[54], [55], [56], [57], [58]]. Only one qualitative study noted a lack of tool satisfaction; however, it was with physicians. In that study, while the patients expressed appreciation for the tool, the non-palliative care providers reported low satisfaction rates, discussing concerns that the tool would devalue their role while also providing the patients a “death message” [57].

## 4 Discussion and conclusion

### 4.1 Discussion

Our research suggests that decision aid use for end-of-life care is viewed favorably by patients and improves outcomes related to patients' decision certainty, knowledge, and desires for less aggressive care. Studies across numerous countries report these positive findings, suggesting that these patient-centered tools can provide value across different cultures and health systems. Furthermore, our review shows that using IPDAS guidelines when developing decision aids may result in more effective decision aids for end-of-life care.

There has been a paradigm shift in healthcare over the last couple of decades to a more patient-centered system [59]. With this approach, the patient is a more active participant in their healthcare. The use of decision aids ties in with this transforming approach to care; these tools help align the care received with the patient's values and preferences. Our review provides evidence across studies that decision aids for end-of-life care can effectively accomplish this goal. Therefore, payers, including private and public programs like Medicare, should consider investing in developing and implementing decision aids to help their members choose their dying preferences.

Two of the handful of studies that did not report positive results examined outcomes from the provider perspective [19,57]. In these cases, the providers' viewpoints on the aids did not coincide with their patients. Further research should examine whether providers are observing actual negative consequences for patients and their surrogates or whether these results relate to a reluctance to change practices or some other conflicting viewpoint. While the evidence consistently highlights that decision aids improve patient knowledge of end-of-life choices and increase desires to pursue less aggressive care, whether patients and their surrogates take steps to complete forms that would help ensure meeting patient preferences is unclear. In examining our review findings through the lens of the Stages of Change Model, also called the Transtheoretical Model [60], it appears that decision aids may have more of an impact on the earlier stages of change (pre-contemplation, contemplation, and preparation) than the later “action” stage of change. Future research should investigate strategies for helping patients and their surrogates progress through the action stage of change, where they modify their end-of-life care wishes in writing; this, in turn, would likely increase the chances of receiving their intended end-of-life care experience.

Decision aids for end-of-life care should ideally be tools that the patients and families can revisit. In a longitudinal analysis of a discrete choice experiment, Yong and colleagues [61] found that advanced cancer patients' preferences for quality-of-life outcomes versus survival extension in Malaysia changed over time. After three months, the patients attributed more importance to physical functioning, pain control, and costs and less importance to psychological functioning, social functioning, survival, and place of death than they had initially. Healthcare professionals and researchers who develop decision aids for end-of-life care should consider that patient preferences may change and integrate flexibility and adaptability into the tools.

With all studies reviewed from high-income economies [17], patients and their families and surrogates from lower-income countries are not represented. Future studies must examine decision aids delivered to individuals across the globe. People from different cultures can respond to decisions about death in various ways [62], and decision aids for one cultural group may not have the same effectiveness for a group from a separate region of the world or even another area of the same country.

### 4.2 Innovation

Opting for comfort care instead of life-sustaining treatment often happens despite the patient not intending that choice [63] or patients and their families not being aware of palliative care and its benefits [64]. Our review is innovative in that we examine all studies that investigated the effectiveness of decision aids for end-of-life care to determine which decision aids may work to potentially prevent the unintended outcome that happens too frequently - pursuing life-sustaining treatment that results in unnecessary patient suffering. With the recent advent of available machine learning technology, there is an opportunity to build upon the effective decision aids identified in this review to enhance decision aids in the future. Lamanna and Byrne [65] suggest that an autonomy algorithm could help predict patient preferences using health records and other patient characteristics. Our review finding that following IPDAS guidelines during decision aid development is associated with positive end-of-life care outcomes reinforces the importance of incorporating these guidelines when using innovative technologies such as machine learning in developing decision aids.

### 4.3 Limitations

By limiting our study to peer-reviewed research, we may be missing case studies or reports evaluating end-of-life decision aids. The inclusion criteria required studies to explicitly highlight an end-of-life care focus of the decision aid. As such, research on comprehensive disease-specific decision aids which may have an end-of-life care arm were not included in the scope of this review. In addition, by summarizing the themes of qualitative research, certain nuances related to specific decision aids were not included. Similarly, our process of categorizing quantitative outcomes and summarizing success simplified complex statistical findings related to the decision aids studied.

### 4.4 Conclusion

Our systematic review highlights the vital role decision aids can play in improving the end-of-life experience for patients and their families and surrogates. While there are many effective decision aids, implementing them in practice and before it's too late for patients should be a focus of future research. As researchers and healthcare professionals work to improve the quality of life for dying individuals, using effective decision aids can contribute to patient-centered care, promoting dignity and respect at a most challenging stage of life.

## Funding

This research did not receive any specific grant from funding agencies in the public, commercial, or not-for-profit sectors.

## CRediT authorship contribution statement

**M. Courtney Hughes:** Writing – review & editing, Writing – original draft, Supervision, Methodology, Formal analysis, Data curation, Conceptualization. **Erin Vernon:** Writing – review & editing, Writing – original draft, Supervision, Methodology, Formal analysis, Data curation, Conceptualization. **Chinenye Egwuonwu:** Writing – original draft, Formal analysis, Data curation. **Oluwatoyosi Afolabi:** Writing – original draft, Formal analysis, Data curation.

## Declaration of competing interest

The authors declare that they have no known competing financial interests or personal relationships that could have appeared to influence the work reported in this paper.

## Appendix A Supplementary data

Supplementary material Image 1
