# Supplementary material for: Measuring decision aid effectiveness for end-of-life care: A systematic review
Source: PEC Innov. 2024 Mar 13;4:100273. doi: 10.1016/j.pecinn.2024.100273 (PMC10957449; doi:10.1016/j.pecinn.2024.100273)
Supplement: Supplementary file 1 — Supplementary material [file mmc1.docx]

**Supplementary Tables**

**Supplementary Table 1:** Quantitative analysis articles

| **First Author (Year)** | **Decision Aid Name** | **Decision Aid Format** | **Study Disease**  **Focus** | **Study**  **Design** | **Comparator** | **Outcome Measure (Success Y/N/nd)** | **Outcome Category** | **Study Quality** |
| --- | --- | --- | --- | --- | --- | --- | --- | --- |
| Green (2009), USA | Making Your Wishes Known: Planning Your Medical Future | Online Interactive | General end of life and cancer patients | Survey | None | High program satisfaction ratings (Y) | Tool satisfaction | Low |
| Volandes (2009), USA | Unnamed 2 minute video support tool of advanced dementia patient to supplement verbal narratives | Video | Advanced dementia | RCT | Verbal narrative without decision support tool | Concordant preferences between patients and their surrogates (Y) | Communication improvements | Medium |
| Ozanne (2009), USA | Living with Metastatic Breast Cancer: Making the Journey Your Own | Video & Booklet | Metastatic Breast Cancer | Survey | Pre-post | Influence on discussions about advance directives (N). | Communication improvements | Medium |
| Volandes (2010), USA | Video support tool of advanced dementia patient to supplement verbal narratives (no official name) | Video | Advanced Dementia | Survey | Pre-post | Decreased uncertainty regarding subjects’ preferences (Y) | Decisional conflict | High |
| Hossler (2011), USA | Making Your Wishes Known: Planning Your Medical Future | Online interactive | Amyotrophic Lateral Sclerosis (ALS) | Survey | Pre-post | Decision aid satisfaction (Y); Patient sense of self determination (nd) | Tool satisfaction; Decisional conflict | Low |
| Volandes (2011), USA | Video support tool of advanced dementia patients regarding goals of care (no official name) | Video | Advanced Dementia | RCT | Verbal description of advanced dementia and goals of care without video | Comfort care selection (Y) | Less aggressive care desired | High |
| Yun (2011), South Korea | Patients Want to Know the Truth | Video & companion workbook | Terminally ill cancer patients | RCT | Korean version of a US National Cancer Institute DVD of similar length on pain management entitled Controlling Cancer Pain: A Video for Patients and Families16 and 29-page educational book on pain control by the Korean Ministry of Health and Welfare entitled Cancer Pain Can Be Controlled. | Decision to discuss terminal prognosis (nd); Decisional conflict and satisfaction (Y); Mood and quality-of-life outcomes (Y); | Communication improvements; Decisional conflict; Patient anxiety or well-being | High |
| Smith (2011), USA | A concise review of diagnosis, prognosis, treatment options, side effects, and when to call the doctor (no official name) | Tables of information | Advanced Cancer (Breast, Colorectal, Lung, and Hormone-Refractory Prostate Cancer) | Survey | Pre-post | Patient finding information helpful (Y) | Tool satisfaction | Low |
| Volandes (2012), USA | Video portraying the three choices of health care in advanced cancer (no official name) | Video | Advanced Cancer | Survey | Pre-post | Knowledge about goals of care (Y), Wanting CPR or ventilation (Y); Documented DNR order (nd) | Knowledge improvements; Less aggressive care desired; Less aggressive care action completed | Medium |
| Kerstholt (2012), the Netherlands | Choice Help (“Keuzehulppz”) | Interactive videos | Healthy individuals | Survey | Only written information provided | Consistency in home death location selection during follow up (Y) | Decisional conflict | Medium |
| Einterz (2014), USA | Goals of Care | Video & Print | Advanced Dementia | Interviews (Surrogate decision makers) | Pre-post | Surrogate-health care provider concordance on primary goal of care (Y); Surrogate involvement in decision making (Y) | Communication improvements | Low |
| Matlock (2014), USA | Looking Ahead: Choices for Medical Care When You’re Seriously Ill | DVD and booklet | Terminal Illness | RCT | Standard palliative care consultation | Decision conflict (nd); Knowledge (nd); Feeling of empowerment (Y) | Decisional conflict; Knowledge improvement | Medium |
| Markham (2015), USA | Making Your Wishes Known: Planning Your Medical Future | Online Interactive | General End-of-Life | Survey | Pre-post | ACP knowledge (Y); Sense of self determination (Y) | Knowledge improvements; Decisional conflict | Low |
| Green (2015), USA | Making Your Wishes Known: Planning Your Medical Future | Online Interactive | Advanced Cancer | RCT | Education materials provided with online advance directive form | ACP Knowledge (Y); Decline in hope (nd); Anxiety (nd) | Knowledge improvements; Decisional conflict | High |
| El-Jawahri (2015), USA | Video regarding CPR and intubation (no official name) | Video | Terminal Illness | RCT | Usual care | Not want CPR (Y); Not want intubation (Y); Have orders to withhold CPR and intubation by hospital discharge (Y);, Documented discussions about preferences (Y) | Less aggressive care desired; Less aggressive care action completed | High |
| Hanson (2015), USA | Goals of Care | Video & structured discussion with provider | Advanced Dementia | RCT | Family decision makers receive informational video on interaction with someone with dementia and a usual care plan meeting with staff | Quality of general and EoL communication (Y); Goal concordance with providers at 3 months (nd); Goal concordance with providers at 9 months or death (Y) | Communication improvements | High |
| Van Scoy (2016), USA | Making Your Wishes Known: Planning Your Medical Future | Online Interactive | Advanced heart failure and chronic obstructive pulmonary disease | Survey | Pre-post | ACP Knowledge (Y); Low decisional conflict (Y); Tool satisfaction (Y) | Knowledge improvements; Tool satisfaction; Decisional conflict | Medium |
| Levi (2017), USA | Making Your Wishes Known: Planning Your Medical Future | Online Interactive | Amyotrophic Lateral Sclerosis (ALS) | Hypothetical clinical vignette questionnaires | Pre-post | Concordance between patient wishes and provider decisions (Y); ACP knowledge (Y); Anxiety (nd) | Communication improvements; Knowledge improvements  ; Patient anxiety or well-being | Medium |
| Bakitas (2017), USA* | Looking Ahead: Choices for Medical Care When You're Seriously Ill | DVD and booklet | Advanced cancer | Closed ended interview questions | None | Satisfaction with format (Y); Would recommend to others (Y) | Tool satisfaction | Low |
| Kapell Brown (2018), Canada | CPR-VDA | Video | End Stage Renal Disease | Survey | Pre-post | CPR knowledge (Y); Decisional self-efficacy (Y); Decision to not want CPR (Y) | Knowledge improvements; Decisional conflict; Less aggressive care desired | Medium |
| Lum (2018), USA | PREPARE plus Advance Directive | Online Interactive | General EoL | RCT | Advance Directive only group | Engaged in ACP discussions and documentation (Y) | Less aggressive care action completed | High |
| Schubart (2019), USA | Making Your Wishes Known: Planning Your Medical Future | Online Interactive | Advanced cancer | RCT | Standard ACP | Physician awareness or adherence (nd); Type of end-of-life-care received (nd) | Communication improvements | High |
| Dharmarajan (2019), USA | PRT Video Tool (no official name) | Video | Advanced cancer | Survey | Pre-post | Reduced decisional uncertainty (Y); Treatment knowledge (Y); Treatment readiness (Y);. Palliative care consultation readiness (nd) | Decisional conflict; Knowledge improvements; Less aggressive care action  completed | Medium |
| Kang (2020), South Korea | Advance Care Planning | Video | General | RCT | 13-page brochure entitled, Understanding the Life-Sustaining Treatment Act, which was developed by the Korean Ministry of Health and Welfare | Intention to document ACP (Y); Palliative care knowledge (Y) | Less aggressive care desired; Knowledge improvements | High |
| Gallegos (2020), USA | Video regarding Physician Orders for Life-Sustaining Treatment (no official name) | Video | General | Survey | Pre-post | Knowledge of POLST form options (Y); Decision satisfaction (Y); Decisional conflict reduction (Y) | Knowledge improvements; Decisional conflict | Medium |
| Lipnick (2020), USA* | Making Your Wishes Known: Planning Your Medical Future | Online interactive | General serious illness | RCT | Standard online advance directive that included a living will form and the opportunity to designate a spokesperson | Presence of stress reported by spokesperson (nd) (Results were the same for patient/spokesperson dyads and patients alone ) | Patient anxiety or well-being | High |
| Hsieh (2020), Taiwan | Virtual reality video (no official name) | Virtual reality video | General | RCT pre-post | No decision aid | Change over time in preference of refusing life-sustaining treatment (Y) | Less aggressive care desired | High |
| Green (2020), USA | Making Your Wishes Known: Planning Your Medical Future | Online interactive | Stage IV cancer | RCT | No decision aid | Number of life-sustaining medical treatments (Y) | Less aggressive care action completed | High |
| Smith (2020), USA | Four Conversations | Online | Metastatic breast cancer | RCT | No decision aid | Change in decisional conflict scores (nd) | Decisional conflict | High |
| Agarwal (2020), USA* | Person-Centered Oncologic Care and Choices (P-COCC) | Video and interview | Advanced gastrointestinal cancer | Survey | Video only and no decision aid combined | Change in personal values (Y) | Decisional conflict | High |
| Thiede (2021), USA* | Making Your Wishes Known: Planning Your Medical Future | Online interactive | General serious illness | RCT | Standard online advance directive that included a living will form and the opportunity to designate a spokesperson | Patient’s spokesperson preparedness for decision making (N); Patient’s spokesperson having engaged conversations with patient and medical team (Y) | Communication improvements | High |
| Ke (2021), Taiwan | Advance care planning brochure (no official name) | Paper | General EoL | Quasi-experimental pre-post | No decision aid | Improvement in congruence between patient and their surrogate (Y) | Communication improvements | High |
| Friend (2021), Japan, USA | Advance directive for artificial nutrition and hydration (AD for ANH) (no official name) | Online interactive | General | Survey | None | Preference for no artificial nutrition and hydration at the end of life (Y) | Less aggressive care desired | Medium |
| Vigouret-Viant (2022), France | Decision Aid Form (DAF) (no official name) | Paper | Cancer | Cohort | None | Planned stratification of care (Y); Pursuing less aggressive care (nd) | Less aggressive care desired; Less aggressive care action completed | Medium |
| Ufere (2022), USA | Advance care planning video (no official name) | Video | Advanced liver disease | RCT | Control group who listened to the end-of-life care choices read aloud with no video with visual images | Mean knowledge scores (Y); Less likely to receive CPR (Y); Patients reported being very comfortable watching the video (Y) | Knowledge improvements; Less aggressive care desired; Tool satisfaction | High |
| van Baal (2022), Germany | Supportive and Palliative Care Indicators Tool (SPICT-DE™) | Online information sheet | Chronic, progressive disease | Cohort | Pre-post | Primary care physician’s self‑assessed quality of end-of-life care improvement (Y)** | Patient anxiety or well-being | High |
| Yamamoto (2022), Japan* | Patient decision aids before high risk surgery (no official name) | Booklets | High-risk surgery | Pre-post | None | (Insufficient sample size of 7) Patient’s decision satisfaction; Perception of the need to discuss ACP before surgery; Confidence in proxy decision-making |  | Low |
| Yun (2019), South Korea | Patients Want to Know the Truth | Video and booklet | Advanced cancer | RCT | Cancer pain control group | Less preference for active and life-prolonging treatment (Y); Greater preference for hospice care (Y) | Less aggressive care desired | High |
| Heyland (2020), Canada | Plan Well Guide | Online interactive and paper | General seriously ill | RCT | No decision aid | Stated goals of care (Y); Goal-concordant care (Y); Patient-reported lower decisional conflict (Y); Physician-reported lower decisional conflict (nd) | Decisional conflict; Communication improvements | High |

*Studies with both qualitative and quantitative methods.

**** There was a simultaneous public campaign to inform and connect regional health care providers and stakeholders in end-of-life care

*Notes*: ACP = Advance care planning; Y = yes; N = no; nd = no difference

**Supplementary Table 2:** Qualitative analysis articles

| **First Author (Year)** | **Decision Aid Name** | **Decision Aid Format** | **Study Disease**  **Focus** | **Method (Participants)** | **Outcome Examined** | **Themes** | **Study Quality** |
| --- | --- | --- | --- | --- | --- | --- | --- |
| Jones (2015), USA | Looking Ahead: Choices for medical care when you're seriously ill | DVD and booklet | General seriously ill and healthy | Focus groups (providers, patients) | Tool Satisfaction | Non-palliative care providers saw the decision aid as devaluing the role of the provider. They were also concerned it could be seen as handing patients a “death message.” Patients (seriously ill and healthy) found the decision aid useful and stated it should be introduced sooner rather than later. They believed the decision aid increased patient empowerment | High |
| Uhler (2015), USA | InformedTogether | Online | Chronic Obstructive Pulmonary Disease | Interviews (patients, physicians) | Tool satisfaction; Knowledge improvements | Patients and physicians found InformedTogether acceptable and would recommend that physicians use it with COPD patients; Many patients had difficulty understanding the icon arrays used to communicate estimated prognoses and could not articulate the definitions of the two treatment choices—Full Code and Do Not Resuscitate (DNR); Patients and physicians indicated there should be more information about the implications of the treatment choices | High |
| Bakitas (2017), USA* | Looking Ahead: Choices for Medical Care When You're Seriously Ill® | DVD and booklet | Advanced cancer | Interviews  (patients, caregivers) | Tool satisfaction; Knowledge improvements; Less aggressive care desired | Participants reported 1) a high degree of satisfaction with the decision aid format, as well as with its length and clarity, 2) “the earlier the better” to view it, and 3) feeling empowered, aware of different options, and an urgency to participate in advance care planning | High |
| Jones (2018), USA | DecisionKEYS for Balancing Choices: Cancer Care | Audio CD, decision balance sheet, and booklet | Advanced prostate cancer | Interview  (patients, their decision partners) | Knowledge improvements; Less aggressive care desired | The decision aid facilitated understanding of treatment options; Quality of life was more important than quantity of life; Contact with healthcare providers greatly influenced decisions | High |
| Lipnick (2020), USA* | Making Your Wishes Known: Planning Your Medical Future | Online interactive | General serious illness | Interview (patients, patient spokesperson) | Communication improvements; Knowledge improvements; Decisional conflict | The following impacted spokesperson stress: “1) the nature of the relationship with their loved one, 2) self-described personality and belief systems, 3) knowledge and experience with illness and ACP conversations, 4) attitude toward ACP conversations, and 5) social support in caregiving and decision making” | High |
| Agarwal (2020), USA* | Person-Centered Oncologic Care and Choices (P-COCC) | Video and interview | Advanced gastrointestinal cancer | Interview (patients) | Decisional conflict; Less aggressive care desired; Knowledge improvements; Communication improvements | “1) Participants selected end-of-life treatment options that aligned with their personal values and preferences; 2) Participants expressed negative emotions to life-prolonging care; 3) the video provided most participants with sufficient information to make informed end-of-life care decisions; 4) participants expressed trust in their oncology teams; 5) concerns about illness and prognosis drove a desire for open communication and being understood; 6) relationships with loved ones provided meaning to participants’ lives; 7) religion and spirituality enhanced inner strength and well-being; and 8) participants valued maintaining an identity, keeping agency, and living life outside of having cancer.” | High |
| Thiede (2021), USA* | Making Your Wishes Known: Planning Your Medical Future | Online interactive | General serious illness | Interview (Patients with advanced illness and their spokesperson) | Less aggressive care desired; Decisional conflict; Communication improvements | Patient spokespersons’ perceived preparedness related to 1) perceptions about ACP, 2) level of comfort with uncertainty, 3) relational issues, and 4) personal characteristics; Patient spokespersons believed their knowledge of patient wishes, as well as other personal, relational, situational, and emotional factors would influence their surrogate decisions | High |
| Yamamoto (2022), Japan* | Patient decision aids before high risk surgery (no official name) | Booklets | High-risk surgery | Survey  (Patients) | Communication improvements | Patients reported that using the decision aids provided an opportunity to share their thoughts with their families and inspired them to start mapping their life plans; Patients wanted to share and discuss their decision-making process with medical professionals after the surgery | Low |
| Simmons (2022), USA | Making Your Wishes Known: Planning Your Medical Future | Online Interactive | General serious illness | Interview (patients and their spokesperson) | Decisional conflict; Communication improvements | “ACP helped participants: 1) express clear end-of-life wishes, 2) clarify values, and 3) recognize challenges associated with applying those wishes in complex situations. Shortcomings of ACP included 1) unknown prognostic information or quality-of-life outcomes to inform decision-making, 2) skepticism about patients’ wishes, and 3) complicated emotions impacting end-of-life discussions.” | High |

*Studies with both qualitative and quantitative methods.

*Notes*: ACP = Advance care planning
